# Comparing MicroRNA Profilings of Purified HER-2-Negative and HER-2-Positive Cells Validates miR-362-5p/Sema3A as Characteristic Molecular Change in Triple-Negative Breast Cancers

**DOI:** 10.1155/2019/6057280

**Published:** 2019-12-16

**Authors:** Xiaoqing Zhang, Qi He, Leiqin Sun, Yanfei Zhang, Shengying Qin, Junwei Fan, Jianfeng Wang

**Affiliations:** ^1^Department of Pharmacy, The International Peace Maternity and Child Health Hospital, School of Medicine, Shanghai Jiao Tong University, Shanghai 200030, China; ^2^Department of Breast, The International Peace Maternity and Child Health Hospital, School of Medicine, Shanghai Jiao Tong University, Shanghai 200030, China; ^3^Bio-X Institutes, Key Laboratory for the Genetics of Developmental and Neuropsychiatric Disorders (Ministry of Education), Shanghai Jiao Tong University, Shanghai 200030, China; ^4^Department of General Surgery, Shanghai General Hospital, Shanghai Jiao Tong University School of Medicine, Shanghai 200080, China

## Abstract

**Background:**

HER-2 is a key molecule serving as the therapeutic target, prognostic biomarker, and classification marker in breast cancer. Accurate microRNA profilings had not been conducted in purified tumor cells of HER-2-negative and HER-2-positive tissue specimens obtained from breast cancer patients.

**Methods:**

(i) Differential expression microRNA discovery using laser capture microdissection- (LCM-) assisted specimen preparation and microRNA array chips on HER-2 overexpressing and triple-negative breast carcinoma (TNBC) subtype tissues, (ii) differential expression microRNA validation by quantitative real-time PCR, and (iii) independent validation on tissue microarray.

**Results:**

Five microRNAs (miR-20a-5p, miR-221-3p, miR-362-5p, miR-502-3p, and miR-222-3p) were screened and validated as upregulated microRNAs in TNBC cells comparing to HER-2 overexpressing cells using a microRNA array (5 cases in each group) and quantitative real-time PCR (20 cases in each group). The expression difference of miR-362-5p had the most significant statistical significance (*p* = 0.0016) among the five microRNAs. The expression of miR-362-5p and its target gene Sema3A was further analyzed using in situ hybridization (ISH) and immunohistochemistry on standard tissue sections (*n* = 150). 70.8% of HER-2-negative cells showed moderate expression of miR-362-5p whereas 20.4% HER-2-negative cells correlated with strong expression of miR-362-5p (*p* < 0.0001). The proportion of patients with moderate/strong miR-362-5p expression in luminal, HER-2 overexpressing, and TNBC subtypes were 53.2%, 22.2%, and 74.3%, respectively (*p* = 0.0002). High miR-362-5p expressers had shorter overall survival in the univariate analysis (*p* = 0.046). There was a significant negative correlation between miR-362-5p and Sema3A expression (*p* < 0.0001). The patients with negative/weak Sema3A protein expression had poorer prognosis than those with moderate (HR: 3.723, *p* = 0.021) or strong (HR: 3.966, *p* = 0.013) Sema3A protein expression in the multivariate analysis.

**Conclusions:**

miR-362-5p/Sema3A might provide a promising therapeutic pathway and represents a candidate therapeutic target of the TNBC subtype.

## 1. Introduction

Breast cancer is the highest cancer incidence and the leading cause of cancer-related mortality in women worldwide [1]. The burden of breast cancer is increasing in China; there are more than 1.6 million new cases and 1.2 million people dying of the disease each year [2]. It is well known that breast cancer is a heterogeneous disease consisting of patients with different clinical, pathologic, and molecular characteristics. Currently, the molecular classification provides crucial information of treatment selection and prognostic estimation [3].

Estrogen receptor (ER), progesterone receptor (PR), and human epidermal growth factor receptor 2 (HER-2) are routinely available in breast cancer specimens in routine clinical work without additional tests. ER/PR/HER-2 subtypes classified breast cancer patients into 8 groups: ER^+^/PR^+^/HER-2^−^, ER^+^/PR^+^/HER-2^+^, ER^+^/PR^−^/HER-2^−^, ER^+^/PR^−^/HER-2^+^, ER^−^/PR^+^/HER-2^−^, ER^−^/PR^+^/HER-2^+^, ER^−^/PR^−^/HER-2^−^, and ER^−^/PR^−^/HER-2^+^. According to the analyzing result of 123,780 cases of stage 1-3 primary female invasive breast cancer from the California Cancer Registry, HER-2 overexpressing (ER^−^/PR^−^/HER-2^+^) and triple negative (ER^−^/PR^−^/HER-2^−^) had poorer prognosis than other subtypes. The surrogate classification according to the ER/PR/HER-2 status provided clear separation on the outcome of patients [4–6]. ER/PR/HER-2 subtypes had been an important part of the 8th Edition AJCC Staging Manual, the latest breast cancer staging guidelines [7].

Triple-negative breast cancer (TNBC) had the poorest prognosis and accounts for approximately 15%-20% of all breast cancers. Patients with triple-negative subtype had a significantly increased risk of tumor recurrence and mortalities after adjuvant therapy [8]. The poor prognosis of patients with TNBC was possibly due to the lack of effective therapeutic target. Many studies were focusing on discovering actionable molecular targets to treat patients with these tumors. Germline BRCA1/2 mutations or “BRCAness” was one of the molecular features of TNBC partly responsible for important elements of biological behavior including high proliferative activity, an increased immunological infiltrate, a basal-like and mesenchymal phenotype, and deficiency in homologous recombination. The difference of protein expression, mRNA signatures, and genomic alterations between TNBC and another subtype of breast cancer remains unclear. Functional omics research could identify potentially actionable molecular features of TNBC [9, 10].

HER-2-overexpressing cancer often had a highly aggressive phenotype and was associated with metastasis to the lymph nodes and distant organs. Using anti-HER-2 antibodies as a molecular target-based therapy might ameliorate the prognosis of HER-2-overexpressing breast cancer [11]. HER-2 protein overexpressing due to gene amplification derived oncogenic signaling in adenocarcinomas of various organs and tissues of origin including esophagogastric, breast, ovarian, pancreatic, colorectal, and uterine [12]. We focused on the impact of HER-2 expression on microRNA in clinical breast cancer samples excluding the effects of confounding factors of ER and PR expression.

MicroRNAs are a class of noncoding endogenous RNA molecules (containing about 22–25 nucleotides long), which function via base-pairing with complementary sequences within mRNA molecules, leading to their translational repression or degradation. Dysregulation of the microRNA profile has been associated with extensive cell pathological processes underlying progression involving the development and progression of various human malignancies [13–15]. A meta-analysis showed that the key players for ER, PR, and HER signaling pathways are under the control of several microRNAs which could classify breast cancer subtypes [16]. There was growing evidence that specific microRNAs might be of clinical value as both predictive markers and potential therapeutic targets in TNBC [17].

As we know, tumor tissue consists of epithelial cells and mesenchymal cells which could lead to the result bias of high throughput screening technology. Laser capture microdissection (LCM) is used to isolate pure cell populations from heterogeneous tumor tissue [18, 19].

In the present study, we firstly compared the microRNA profilings of purifying cancer cells of 2 subtypes of breast cancer, TNBC and HER-2 overexpressing, by coupling LCM and microRNA array technology. miR-362-5p was upregulated in TNBC cells validated by qRT-PCR and in situ hybridization (ISH). The clinical pathological significance of aberrant expression of miR-362-5p and its target gene Sema3A was analyzed.

## 2. Patients and Methods

### 2.1. Human Breast Cancer Tissues and HER-2 Status Determination

25 TNBC and 25 HER-2 overexpressing paraffin tissue samples were obtained from patients who underwent modified radical mastectomy at the General Surgery Department of Shanghai General Hospital. No chemotherapy or radiation therapy was applied to these patients before operation. IHC was carried out by using antibodies against HER-2, ER, and PR proteins (Abcam, Cambridge, MA) to verify the subtypes of every specimen. Five TNBC tumors and five HER-2 overexpressing tumor samples were randomly selected to analyze their microRNA profiles using microRNA array and LCM. All patients provided informed consent, and the study was approved by the institutional review board of Shanghai General Hospital. Furthermore, the HBre-Duc150Sur-02 tissue array which included 150 breast cancer cases was purchased from Outdo Biotech (Shanghai) for verifying the results of the microRNA chip. Tumor staging was conducted according to the American Joint Committee on Cancer (AJCC) sixth edition cancer staging system. This tissue array included 24 stage I, 82 stage II, and 38 stage III cases. The median follow-up period was 83 months (range 2-119 months).

### 2.2. LCM and RNA Extraction

Three-centimeter-thick sections were cut from each of the HER-2-positive and triple-negative tissue specimens, and the thickness of each slice was 10 *μ*m. These slices underwent xylene dewaxing, gradient ethanol hydration, and hematoxylin-eosin stain. Approximately 5 mm^2^ tumor parenchymal cells in each sample were captured using the Veritas LC/LCM system (Arcturus Engineering, Mountain View, CA). Total RNA was extracted and purified using mirVana™ microRNA Isolation Kit (Cat#AM1560, Ambion, Austin, TX, US) following the manufacturer's instructions and checked for a RIN number to inspect RNA integration by an Agilent Bioanalyzer 2100 (Agilent technologies, Santa Clara, CA, US).

### 2.3. Microarray Hybridization, Scanning, and Acquisition of Data

MicroRNA microarray profiling was performed using an Agilent Human microRNA (8^∗^60 K) V19.0 (Santa Clara, CA, USA). MicroRNA molecular in total RNA was labeled by microRNA Complete Labeling and Hyb Kit (Agilent technologies, Santa Clara, CA) following the manufacturer' s instructions for labeling sections. Each slide was hybridized with 100 ng Cy3-labeled RNA using microRNA Complete Labeling and Hyb Kit (Agilent technologies, Santa Clara, CA) in hybridization Oven (Agilent technologies, Santa Clara, CA) at 55°C, 20 rpm for 20 hours according to the manufacturer's instructions for hybridization section. After hybridization, slides were washed in staining dishes (Thermo Shandon, Waltham, MA) with a Gene Expression Wash Buffer Kit (Agilent technologies, Santa Clara, CA). Slides were scanned by an Agilent Microarray Scanner (Agilent technologies, Santa Clara, CA) and Feature Extraction software 10.7 (Agilent technologies, Santa Clara, CA, US) with default settings. Raw data were normalized by Quantile algorithm, Gene Spring Software 11.0 (Agilent technologies, Santa Clara, CA).

### 2.4. Bioinformatics Analysis of MicroRNA Target Gene Prediction

The target genes of differential expression microRNA forecasted by both TargetScan (http://www.targetscan.org/) and Mirdb (http://mirdb.org/) were selected for subsequent analysis. Molecular function categories and the enriched pathway of target genes were explored using GO Enrichment Analysis (http://www.geneontology.org/page/go-enrichment-analysis) and the KEGG PATHWAY Database (https://www.kegg.jp/kegg/pathway.html).

### 2.5. Quantitative Real-Time PCR

Total RNA, including microRNAs, was isolated from tissue specimens using TRIzol reagent (Invitrogen, Carlsbad, CA, USA) according to the manufacturer's protocol. The first strand cDNA was synthesized with the RevertAid First Strand cDNA Synthesis Kit (MBI Fermentas, Vilnius, Lithuania) using 1 *μ*g of total RNA as the template. MicroRNAs were prepared with the High-Specificity microRNA qRT-PCR Detection Kit (Stratagene, Santa Clara, CA, USA), and U6 was used as an endogenous control. Real-time PCR was used to analyze the expression of each microRNA using the ViiA™ 7 system (Thermo Fisher Scientific, Waltham, MA, USA) according to the manufacturer's instructions. The qRT-PCR primers are shown in Additional file 1: [Supplementary-material supplementary-material-1].

### 2.6. In Situ Hybridization (ISH) and Immunohistochemistry on Tissue Microarray

The tissue microarray paraffin block was cut into 4 *μ*m pathology slides. The slides were dewaxed in xylene for 15 min twice and dehydrated by immersion in 100% ethanol for 5 min. Then, the slides were air-dried, incubated with pepsin at 37°C for 15 min, fixed in 4% paraformaldehyde, and dehydrated in 90% ethanol sequentially. The slide was incubated with the digoxigenin-labeled probe (Hs_miR-362-5p_1 miScript Primer Assay, Exiqon, Denmark) complementary to miR-362-5p at 37°C overnight, according to the manufacturer's instructions. The slides were washed twice with 2x saline-sodium citrate buffer at room temperature and incubated with mouse anti-digoxigenin monoclonal antibody according to the manufacturer's protocol.

HBre-Duc150Sur-02 tissue sections 4 *μ*m thick were processed for detection of Sema3A using a rabbit polyclonal antibody anti-Semaphorin 3A antibody ab80011 (diluted 1 : 50; Abcam, Cambridge, United Kingdom) and Goat Anti-Rabbit IgG H&L (HRP) preadsorbed (Abcam, Cambridge, United Kingdom). The sections were then counterstained with Mayer hematoxylin. Two independent investigators scored the sections without knowledge of the patient outcome (double-blinded). The proportion of positively stained tumor cells was graded as follows: 0 (no positive cells), 1 (<10% positive cells), 2 (10–50% positive cells), 3 (>50% positive cells). The intensity of the staining was recorded on a scale of 0 (no staining), 1 (weak staining), 2 (moderate staining), and 3 (strong staining). The staining index (SI) was defined as the proportion of positively stained tumor cells multiplied by staining intensity.

### 2.7. Cell Lines

The human breast cancer cell lines MDA-MB-468 (triple-negative) and SK-BR-3 (HER-2^+^) were obtained from the Type Culture Collection of the Chinese Academy of Sciences (Shanghai, PR China). The cells were cultured under conditions suggested by the vendors.

### 2.8. Western Blot Analysis

Total proteins were extracted from cultured cells using RIPA lysis buffer (Beyotime Biotechnology, Jiangsu, China). Protein concentrations were determined using a BCA protein assay kit (Beyotime Biotechnology, Jiangsu, China). Equal amounts of protein were separated by electrophoresis on SDS polyacrylamide gel and transferred onto polyvinylidene difluoride membranes. The membranes were blocked with 5% nonfat milk solution for 1 h and then incubated with a primary antibody overnight at 4°C and a secondary antibody at room temperature, successively. The bands were detected by ECL chemiluminescence (Millipore, USA) according to the manufacturer's instructions. Expression of actin was applied as the internal control to confirm equal loading of whole protein. The following antibodies were used: anti-Semaphorin 3A antibody (diluted 1 : 50; Abcam, Cambridge, United Kingdom), Goat Anti-Rabbit IgG H&L (HRP) preadsorbed (Abcam, Cambridge, United Kingdom) and anti-actin (Sigma).

### 2.9. Statistics

Statistical analyses were performed using the SPSS statistical software program version 20 (SPSS Inc., Chicago, IL, USA). The *χ*^2^ test or Fisher's exact test for enumeration data was used to analyze the relationship between miR-362-5p and clinicopathological features. The Kaplan-Meier method was used to analyze the survival rates, and the differences between the survival curves were examined by the log-rank test. Univariate and multivariate survival analyses were performed using Cox proportional hazard models. *p* < 0.05 was considered statistically significant.

## 3. Results

### 3.1. Characteristic MicroRNA Expression Profiles of HER-2-Negative and HER-2-Positive Breast Cancer Cells

**Figure 1:**
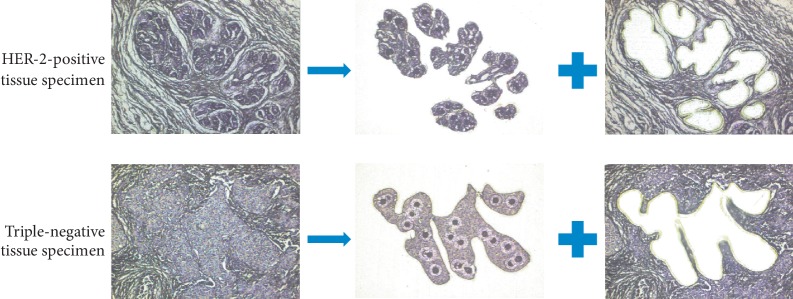
HER-2-negative and HER-2-positive breast cancer cells. Cancer cells were captured from fixed tissue sections by laser capture microdissection.

Parenchymal cells in breast carcinoma tissues were purified using the Veritas LC/LCM system (Figure 1). The amounts of RNA of 5 HER-2-positive specimens were 102.0 ng, 96.0 ng, 126.0 ng, 108.0 ng, and 66.0 ng; The amounts of RNA of 5 HER-2-negative specimens were 114.0 ng, 243.0 ng, 150.0 ng, 141.0 ng, and 135.0 ng. MicroRNA microarray detection rates of 5 HER-2-positive specimens were 28.22%, 27.12%, 22.18%, 30.21%, and 19.57%; microRNA microarray detection rates of 5 HER-2-negative specimens were 33.05%, 34.45%, 31.75%, 34.85%, and 36.09%. Coefficients of variation of microRNA microarray of 5 HER-2-positive specimens were 5.68%, 5.55%, 6.53%, 5.73%, and 8.47%; coefficients of variation of microRNA microarray of 5 HER-2-negitive specimens were 6.63%, 5.42%, 5.59%, 5.55%, and 6.42%. These quality control parameters reflected that a small amount of RNA from paraffin samples in our study was suitable for high throughput microRNA detection.

**Figure 2:**
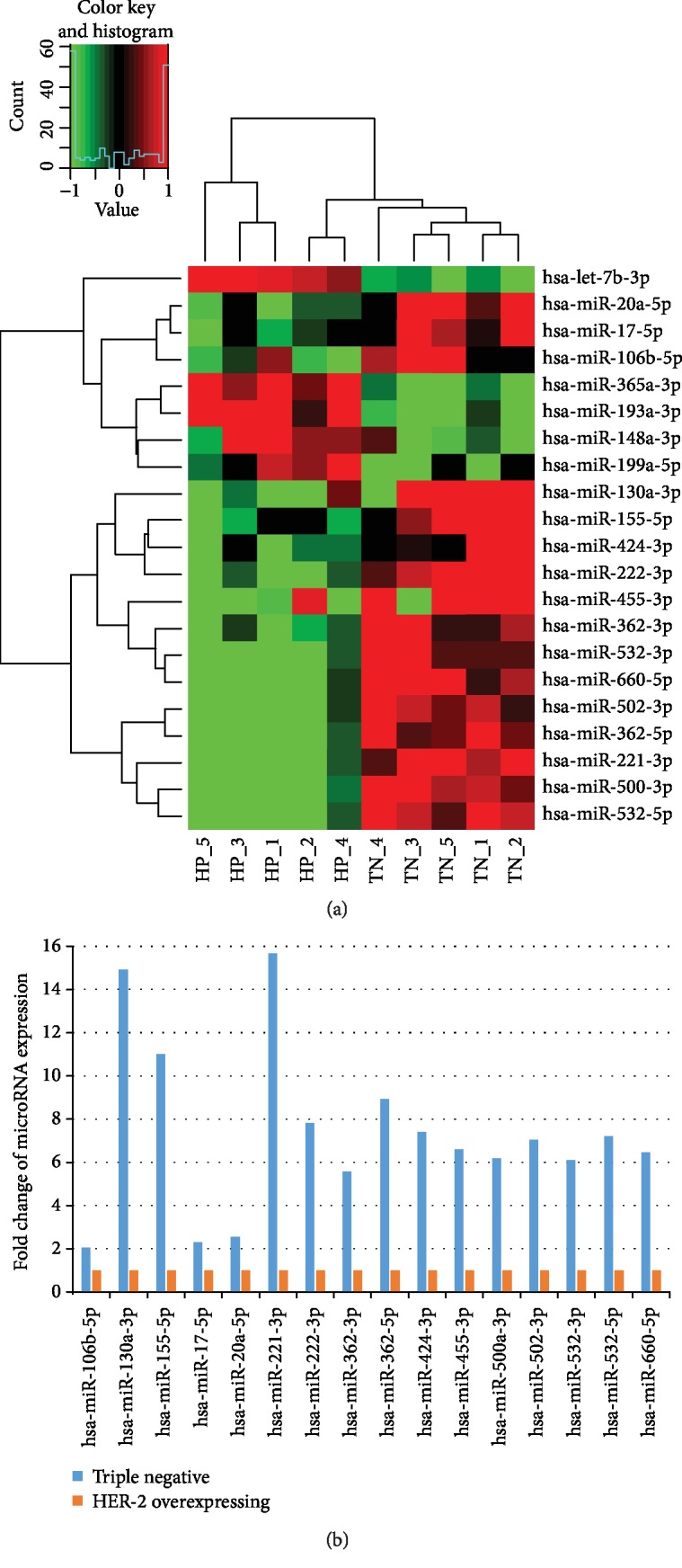
MicroRNA microarray analysis of HER-2-negative and HER-2-positive breast cancer cells. (a) Unsupervised hierarchical clustering analysis of the 21 microRNAs differentially expressed between the HER-2-negative and HER-2-positive breast cancer cells. Higher intensities of red indicate higher expression levels, while lower intensities of green indicate lower expression levels. (b) Fold change of 16 differential expression microRNAs which was upregulated in TNBC cells.

21 miRNAs were found to be significantly differentially expressed and could distinguish effectively HER-2-negative and HER-2-positive breast cancer cells (Figure 2(a)). Filter criteria of differential expression microRNA was that fold change was greater than 2 and *p* value is less than 0.05. The expression of 16 microRNAs in TNBC subtypes cells was higher than that in HER-2-overexpressing cells (Figure 2(b)), and 5 microRNAs were downregulated in TNBC cells.

### 3.2. Functional Categorization and Pathway Analysis of Target Genes of Differential Expression MicroRNAs

**Figure 3:**
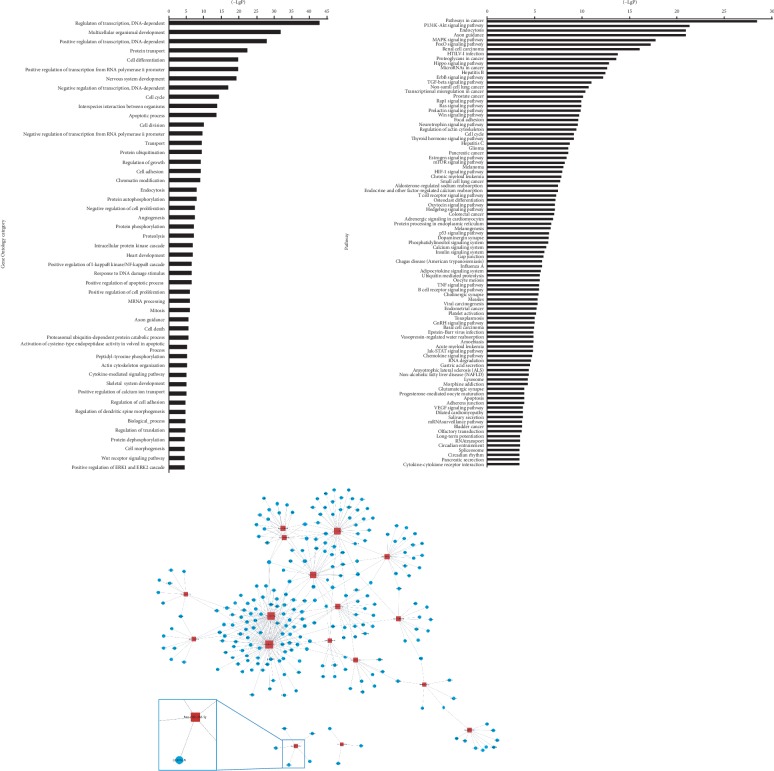
Bioinformatics analysis of microRNA and target gene prediction. (a) Functional annotations of the predicted target genes. Significant GO terms of microRNA targets. The vertical axis represents GO category and the horizontal axis represents the negative logarithm of *p* value (-log *p* value), which indicates the significant level of GOs. (b) Pathway analysis via KEGG. The vertical axis is the pathway category, and the horizontal axis is the negative logarithm of *p* value (-log *p*) that represents the significant level of pathways. (c) GO gene network analysis. The circle represents the gene and the shape of the square represents microRNA, and their relationship was represented by one edge.

The target genes of microRNA were predicated using TargetScan software (http://www.targetscan.org/) and Mirdb (http://mirdb.org/). The intersection of target genes predicated by TargetScan and Mirdb database was used for subsequent analysis. The significant Gene Ontology category involved transcriptional regulation, protein transport, cell differentiation, cell cycle, apoptosis process, protein ubiquitylation, etc. (Figure 3(a)). The pathways of target genes enriched included pathways in cancer, PI3K-Akt signaling pathway, MAPK signaling pathway, microRNA in cancer, and ErbB signaling pathway (Figure 3(b)). A microRNA-gene net rated to HER-2 status in breast cancer cells was built based on GO analysis and Pathway analysis (Figure 3(c)).

### 3.3. Confirmation of Individual MicroRNA Expression in the MicroRNA Microarray Data

**Figure 4:**
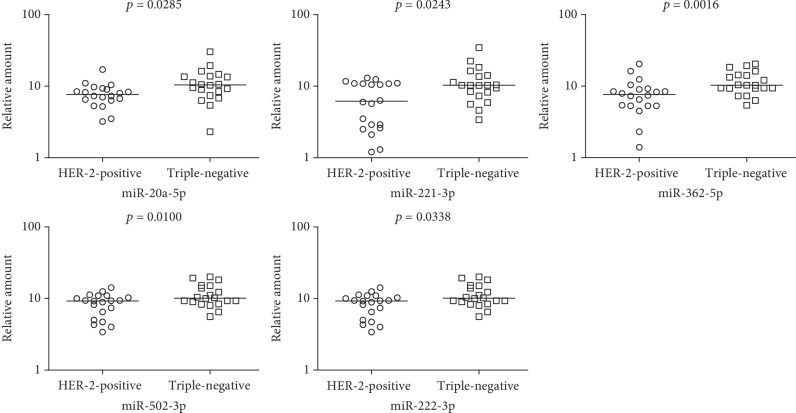
Validation of the 5 upregulated microRNAs screened by microRNA microarray using qRT-PCR. The expressions of miR-20a-5p, miR-221-3p, miR-362-5p, miR-502-3p, and miR-222-3p were significantly higher in HER-2-negative cells compared with HER-2-positive cells.

Five microRNAs (miR-20a-5p, miR-221-3p, miR-362-5p, miR-502-3p, and miR-222-3p) were validated as upregulated microRNAs in TNBC cells in independent cancer samples (20 cases in each group) by quantitative real-time PCR (Figure 4). The expression difference of miR-362-5p had the most significant statistical significance (*p* = 0.0016).

### 3.4. Expression of miR-362-5p and Its Target Gene Sema3A

**Figure 5:**
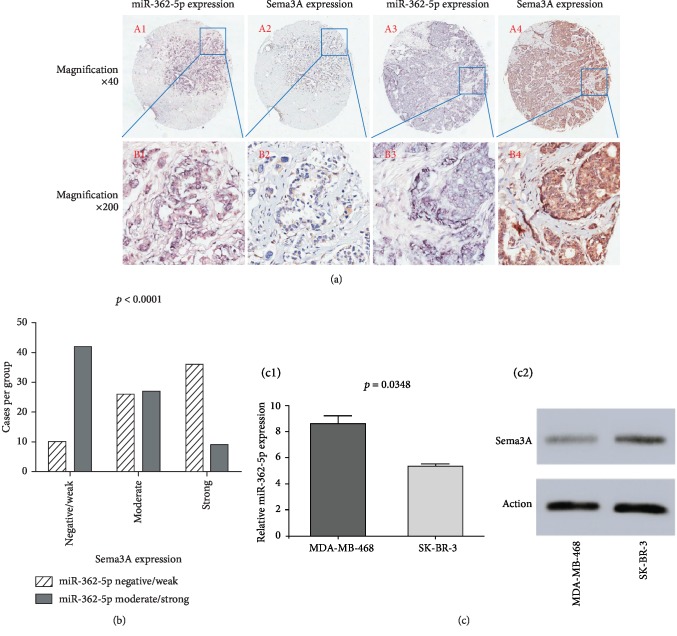
Representative images from in situ hybridization and immunohistochemistry both in the same location of tissue microarray (a). miR-362-5p expression was significantly negatively correlated with the Sema3A expression (b). Relative miR-362-5p expression in the MDA-MB-468 cell line (triple-negative) and SK-BR-3 cell line (HER-2^+^) assessed by quantitative real-time PCR analysis; experiments were repeated three times (C1); Sema3A protein expression in the MDA-MB-468 cell line and SK-BR-3 cell line was assessed by detection by Western blot (C2).

48% (72/150) of breast cancer cases in HBre-Duc150Sur-02 tissue array showed negative and weak miR-362-5p expression and 52 (78/150) cases showed moderate and strong miR-362-5p expression. The percentage of people with negative/weak, moderate, and strong Sema3A protein expression was 34.7 (52/150), 35.3 (53/150), and 30% (45/150), respectively. There was a significant negative correlation between miR-362-5p and Sema3A expression (Figures 5(a) and 5(b)) (*p* < 0.0001).

miR-362-5p expression in the MDA-MB-468 cell line (triple-negative) was significantly higher than that in SK-BR-3 (HER-2^+^) (*p* = 0.0348, Figure 5(c), C1). Sema3A protein expression in the MDA-MB-468 cell line was lower than that in SK-BR-3 (Figure 5(c), C2).

**Figure 6:**
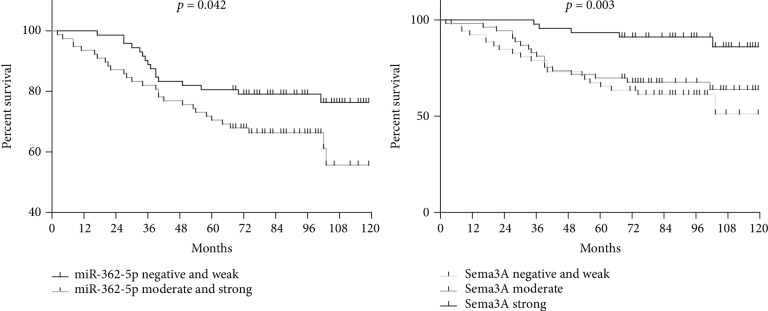
Kaplan-Meier analysis of overall survival (OS) based on the expression of miR-362-5p and Sema3A.

The moderate and strong expression rates of miR-362-5p were 70.8% and 20.4% in HER-2-negative cells, respectively, with a significant difference between the two groups (*p* < 0.0001). The proportion of patients with moderate and strong miR-362-5p expression in luminal, HER-2 overexpressing, and TNBC subtypes was 53.2%, 22.2%, and 74.3%, respectively (*p* = 0.0002) (Table 1). High miR-362-5p expressers had shorter overall survival in the univariate analysis (*p* = 0.042) (Figure 6).

The negative and weak expression rate of Sema3A protein was 16.7%, 34.1%, and 44.7% in AJCC stage I, stage II, and stage III, respectively, with a significant difference between the 3 groups (*p* = 0.0407) (Table 1). Sema3A protein was an independent prognostic factor for breast cancer patients in the HBre-Duc150Sur-02 tissue array (Table 2).

## 4. Discussion

HER-2 is a key molecule serving as the therapeutic target, prognostic biomarker, and classification marker in breast cancer. Comparing the microRNA profiling of HER-2-negative and HER-2-positive cells was helpful to understand the molecular mechanism and screen the therapy targets of breast cancer [20–22]. Three measures to improve the accuracy of experimental data were made in the present study: first, specific microRNA expression profiling of TNBC and HER-2-overexpressing breast cancer was constructed to ruled out the confounding factors of ER and PR expression states [23–26]; secondly, tumor parenchymal cells were separated and purified by LCM technology for excluding the bias resulting from microRNA expression of stromal cells [27, 28]; thirdly, we investigated the cells come from cancer tissues rather than cell lines because the tumor microenvironment leads to the altered microRNA expression. The expression of 16 microRNAs in TNBC subtype cells was higher than that in HER-2-overexpressing cells, and 5 microRNAs were downregulated in TNBC cells. The target gene of these differential microRNA expressions was enriched in tumor-related signaling pathways, including microRNAs in cancer and the ErbB signaling pathway. Five microRNAs (miR-20a-5p, miR-221-3p, miR-362-5p, miR-502-3p, and miR-222-3p) were validated as upregulated microRNAs in TNBC cells in independent cancer samples. The expression difference of miR-362-5p had the most significant statistical significance.

Recently, miR-362-5p was reported as an onco-microRNA in various types of solid tumors and hematological malignancies. miR-362-5p overexpression can facilitate cell proliferation, colony formation, and resistance to cisplatin-induced apoptosis in BGC-823 and SGC-7901 gastric cancer cells via repressing the tumor suppressor CYLD and increasing NF-*κ*B activity [29]. miR-362-5p was significantly upregulated in hepatocellular carcinoma (HCC) and involved in HCC progression through CYLD to activate the NF-*κ*B signaling pathway. Suppression of miR-362-5p expression significantly reduced cell proliferation, clonogenicity, migration, and invasion in HCC cell lines as well as tumor growth and metastasis in a liver tumor model [30]. In hematological malignancies, high miR-362-5p expression was associated with poorer overall survival implicating the oncogenic function in acute myeloid leukemia (AML) development [31]; miR-362-5p was upregulated in chronic myeloid leukemia (CML) cell lines and fresh blood samples from CML patients and was associated with growth arrest and DNA damage-inducible (GADD)45*α* downregulation [32].

Current research of miR-362-5p in breast cancer is limited to cell experiments in vitro. miR-362-5p expression was higher in breast cancer MDA-MB-231 and MCF7 cell lines than the control CCD-1095Sk cell line. The downregulated expression of miR-362-5p led to a significant reduction of cell proliferation, migration, and invasion in human breast cancer MCF7 cells, which suggested that miR-362-5p may act as a novel potential therapeutic target for the treatment of breast cancer [33]. In the present study, we firstly validated that the expression of miR-362-5p in HER-2-negative breast cancer cells was higher than that in HER-2-positive cells using microRNA array and qRT-PCR technology. The clinicopathologic role of miR-362-5p in breast cancer is explored by combined tissue array and in situ hybridization. The moderate and strong expression rates of miR-362-5p were 70.8% and 20.4% in HER-2-negative cells, respectively, with a significant difference between the two groups (*p* < 0.0001). The proportion of patients with moderate and strong miR-362-5p expression in luminal, HER-2 overexpressing, and TNBC subtypes was 53.2%, 22.2%, and 74.3%, respectively (*p* = 0.0002). High miR-362-5p expressers had shorter overall survival in the univariate analysis (*p* = 0.042).

MicroRNAs play the key role in the fine-tuning of diverse cellular functions by binding to the 3′ untranslated region of target mRNAs and downregulate its expression [34, 35]. We speculated that miR-362-5p participated in the carcinogenesis and development of breast cancer by regulating its target gene Sema3A. The first reason was that Sema3A was predicted as the target gene of miR-362-5p and the interaction between miR-362-5p and Sema3A appeared in our microRNA-mRNA net. The second reason is that a recent study confirmed that the direct binding of miR-362-5p to the 3′UTR of Sema3A by luciferase reporter assay in non-small-cell lung carcinoma (NSCLC). Also, the negative correlation between miR-362 expression and Sema3A expression was observed in clinical NSCLC tissue samples. Aberration of the miR-362/Sema3A axis might be the molecular mechanism of NSCLC invasion and migration and could lead to a potential therapeutic target in NSCLC treatment [36].

Semaphorins are a large family including at least 30 members which are divided into 3 types: secretory, transmembrane, and GPI-anchored, and 8 subgroups. Sema3A expression is found in the central nervous system and other tissues and functions in the physiological and pathological processes, including axon guidance, cell migration, tumor growth, immune response, and angiogenesis [37]. It was found that class 3 semaphorins (Sema3A, Sema3B, and Sema3F) decreased with the transition from in situ to invasive cancer and Sema3A expression was only significantly reduced once invasion had occurred [38]. Other findings have showed that vascular endothelial growth factor-induced angiogenesis is inhibited by Sema3A in the breast cancer cell line and that Sema3A modulates phosphorylation of PTEN and FOXO3a and expression of MelCAM, leading to suppression of tumor growth and angiogenesis using an in vivo breast cancer mouse model [39]. These research evidences suggested that Sema3A may serve as a candidate tumor suppressor that attenuates breast tumor progression.

Our results showed that the negative correlation between Sema3A protein expression and the AJCC stage (*p* = 0.0407) and the patients with negative/weak Sema3A protein expression had poorer prognosis than those with moderate or strong Sema3A protein expression (*p* = 0.021 and 0.013). We also observed that there are remarkable negative relevant relations between the miR-362-5p expression and Sema3A protein expression (*p* < 0.0001) using in situ hybridization, immunohistochemistry, tissue microarray techniques, and human breast cancer cell lines MDA-MB-468 (triple-negative) and SK-BR-3 (HER-2^+^). This was the first study to investigate the clinical significance of the miR-362-5p/Sema3A axis in breast cancer.

In summary, we present the first report using LCM and a microRNA array for measuring microRNA profilings of purifying cancer cells of 2 subtypes of breast cancer, TNBC, and HER-2 overexpressing. We have identified that miR-362-5p might be a specific high expression microRNA in TNBC subtype breast cancer. miR-362-5p/Sema3A may provide a promising therapeutic pathway and represents a candidate therapeutic target of TNBC subtype breast cancer.

## Figures and Tables

**Table 1:** Association between miR-362-5p expression, Sema3A protein expression, and clinicopathologic characteristics in breast cancer tissue array (*n* = 150).

	Total	miR-362-5p expression		*p*	Sema3A expression			*p*
	(*n* = 150)	Negative/weak (*n* = 72)	Moderate/strong (*n* = 78)		Negative/weak (*n* = 52)	Moderate (*n* = 53)	Strong (*n* = 45)	
Age								
≤50 y	46	22	24	0.9774	13	19	14	0.4822
>50 y	104	50	54		39	34	31	
Grade								
I+II	74	37	37	0.6507	27	24	23	0.6301
III	65	35	30		19	25	21	
T stage								
T1	39	24	15	0.0661	9	15	15	0.1875
T2	90	41	49		32	32	26	
T3	17	5	12		9	6	2	
N stage								
N0	78	39	39	0.8396	24	27	27	0.098
N1	38	18	20		14	12	12	
N2	19	8	11		9	5	5	
N3	13	5	8		4	9	0	
AJCC stage								
I	24	16	8	0.0703	4	11	9	0.0407
II	82	38	44		28	25	29	
III	38	14	24		17	16	5	
HER-2								
Negative	89	26	63	<0.0001	34	31	24	0.3932
Positive	54	43	11		15	20	19	
ER								
Negative	78	35	43	0.4765	32	25	21	0.2583
Positive	71	36	35		20	28	23	
PR								
Negative	91	43	48	0.9981	34	32	25	0.4566
Positive	55	26	29		16	19	20	
Types								
ER^+^ and/or PR^+^	79	37	42	0.0002	25	30	24	0.2278
HER-2^+^/ER^−^/PR^−^	27	21	6		6	10	11	
HER-2^−^/ER^−^/PR^−^	35	9	26		17	10	8	

**Table 2:** Univariate and multivariate Cox proportional hazard models for OS.

	Overall survival (OS)			
	Univariate		Multivariate	
	HR (95% CI)	*p*	HR (95% CI)	*p*
Age				
≤50y	1			
>50y	1.597 (0.870-2.931)	0.131		
Grade				
I+II	1			
III	1.085 (0.572-2.057)	0.802		
T stage				
T1	0.239 (0.088-0.644)	0.005		
T2	0.424 (0.198-0.908)	0.027		
T3	1			
N stage				
N0	0.272 (0.113-0.652)	0.004		
N1	0.351 (0.136-0.907)	0.031		
N2	0.466 (0.163-1.331)	0.154		
N3	1			
AJCC stage				
I	0.340 (0.126-0.916)	0.033	0.466 (0.167-1.302)	0.145
II	0.363 (0.189-0.699)	0.002	0.458 (0.231-0.909)	0.025
III	1		1	
HER-2				
Negative	1			
Positive	1.300 (0.708-2.386)	0.397		
ER				
Negative	2.060 (1.104-3.844)	0.023	1	
Positive	1		1.976 (1.020-3.829)	0.044
PR				
Negative	1.749 (0.898-3.407)	0.1		
Positive	1			
Types				
ER^+^ and/or PR^+^	1			
HER-2^+^/ER^−^/PR^−^	1.902 (0.878-4.122)	0.103		
HER-2^−^/ER^−^/PR^−^	2.122 (1.055-4.269)	0.035		
miR-362-5p				
Negative/weak	1.873 (1.010-3.471)	0.046		
Moderate/strong	1			
Sema3A				
Negative/weak	1		1	
Moderate	4.716 (1.773-12.547)	0.002	3.723 (1.221-11.353)	0.021
Strong	3.619 (1.343-9.752)	0.011	3.966 (1.333-11.803)	0.013

## Data Availability

The data used to support the findings of this study are available from the corresponding authors upon request.

## Supplementary Materials

Supplementary Materials Table S1: the primers used for real-time PCR.

## References

[1] DeSantis C. E., Bray F., Ferlay J., Lortet-Tieulent J., Anderson B. O., Jemal A. (2015). International variation in female breast cancer incidence and mortality rates. *Cancer Epidemiology, Biomarkers & Prevention*.

[2] Fan L., Strasser-Weippl K., Li J. J. (2014). Breast cancer in China. *The Lancet Oncology*.

[3] Rakha E. A., Green A. R. (2017). Molecular classification of breast cancer: what the pathologist needs to know. *Pathology*.

[4] Parise C. A., Caggiano V. (2014). Breast cancer survival defined by the ER/PR/HER2 subtypes and a surrogate classification according to tumor grade and immunohistochemical biomarkers. *Journal of Cancer Epidemiology*.

[5] Parise C. A., Caggiano V. (2017). Risk of mortality of node-negative, ER/PR/HER2 breast cancer subtypes in T1, T2, and T3 tumors. *Breast Cancer Research and Treatment*.

[6] Ma H., Ursin G., Xu X. (2018). Body mass index at age 18 years and recent body mass index in relation to risk of breast cancer overall and ER/PR/HER2-defined subtypes in white women and African-American women: a pooled analysis. *Breast Cancer Research*.

[7] Plichta J. K., Ren Y., Thomas S. M. (2018). Implications for breast cancer restaging based on the 8th edition AJCC staging manual. *Annals of Surgery*.

[8] Curigliano G., Goldhirsch A. (2011). The triple-negative subtype: new ideas for the poorest prognosis breast cancer. *Journal of the National Cancer Institute. Monographs*.

[9] Bianchini G., Balko J. M., Mayer I. A., Sanders M. E., Gianni L. (2016). Triple-negative breast cancer: challenges and opportunities of a heterogeneous disease. *Nature Reviews Clinical Oncology*.

[10] Denkert C., Liedtke C., Tutt A., von Minckwitz G. (2017). Molecular alterations in triple-negative breast cancer—the road to new treatment strategies. *Lancet*.

[11] Mandujano-Tinoco E. A., García-Venzor A., Melendez-Zajgla J., Maldonado V. (2018). New emerging roles of microRNAs in breast cancer. *Breast Cancer Research and Treatment*.

[12] Gerson J. N., Skariah S., Denlinger C. S., Astsaturov I. (2017). Perspectives of HER2-targeting in gastric and esophageal cancer. *Expert Opinion on Investigational Drugs*.

[13] Bracken C. P., Scott H. S., Goodall G. J. (2016). A network-biology perspective of microRNA function and dysfunction in cancer. *Nature Reviews Genetics*.

[14] Petrovic N., Ergün S., Isenovic E. R. (2017). Levels of MicroRNA heterogeneity in cancer biology. *Molecular Diagnosis & Therapy*.

[15] D'Angelo B., Benedetti E., Cimini A., Giordano A. (2016). MicroRNAs: a puzzling tool in cancer diagnostics and therapy. *Anticancer Research*.

[16] Islakoglu Y. O., Noyan S., Aydos A., Dedeoglu B. G. (2018). Meta-microRNA biomarker signatures to classify breast cancer subtypes. *OMICS: A Journal of Integrative Biology*.

[17] Piasecka D., Braun M., Kordek R., Sadej R., Romanska H. (2018). MicroRNAs in regulation of triple-negative breast cancer progression. *Journal of Cancer Research and Clinical Oncology*.

[18] Seclaman E., Narita D., Anghel A. (2019). MicroRNA expression in laser micro-dissected breast cancer tissue samples - a pilot study. *Pathology Oncology Research*.

[19] Fetica B., Balacescu O., Balacescu L., Rus M., Berindan-Neagoe I. (2014). An alternative and sensitive method based on LCM and Q-PCR for HER2 testing in breast cancer. *Cancer Biomarkers*.

[20] Manso L., Sanchez-Muñoz A., Calvo I., Izarzugaza Y., Plata J., Rodriguez C. (2018). Late administration of trastuzumab emtansine might lead to loss of chance for better outcome in patients with HER2-positive metastatic breast cancer. *Breast Care*.

[21] Pernas S., Barroso-Sousa R., Tolaney S. M. (2018). Optimal treatment of early stage HER2‐positive breast cancer. *Cancer*.

[22] Voutsadakis I. A. (2019). HER2 in stemness and epithelial-mesenchymal plasticity of breast cancer. *Clinical & Translational Oncology*.

[23] Howard E. W., Yang X. (2018). MicroRNA regulation in estrogen receptor-positive breast cancer and endocrine therapy. *Biological Procedures Online*.

[24] Liu J., Li X., Wang M. (2018). A miR-26a/E2F7 feedback loop contributes to tamoxifen resistance in ER-positive breast cancer. *International Journal of Oncology*.

[25] Guo C., Fu M., Dilimina Y., Liu S., Guo L. (2018). MicroRNA-10b expression and its correlation with molecular subtypes of early invasive ductal carcinoma. *Experimental and Therapeutic Medicine*.

[26] Chernyi V. S., Tarasova P. V., Kozlov V. V., Saik O. V., Kushlinskii N. E., Gulyaeva L. F. (2017). Search of microRNAs regulating the receptor status of breast cancer in silico and experimental confirmation of their expression in tumors. *Bulletin of Experimental Biology and Medicine*.

[27] Ray M., Ruffalo M. M., Bar-Joseph Z. (2018). Construction of integrated microRNA and mRNA immune cell signatures to predict survival of patients with breast and ovarian cancer. *Genes, Chromosomes & Cancer*.

[28] Hannafon B. N., Ding W. Q. (2019). Functional Role of miRNAs in the Progression of Breast Ductal Carcinoma *in Situ*. *The American Journal of Pathology*.

[29] Xia J. T., Chen L. Z., Jian W. H. (2014). MicroRNA-362 induces cell proliferation and apoptosis resistance in gastric cancer by activation of NF-*κ*B signaling. *Journal of Translational Medicine*.

[30] Ni F., Zhao H., Cui H. (2015). MicroRNA-362-5p promotes tumor growth and metastasis by targeting CYLD in hepatocellular carcinoma. *Cancer Letters*.

[31] Ma Q. L., Wang J. H., Yang M., Wang H. P., Jin J. (2018). miR-362-5p as a novel prognostic predictor of cytogenetically normal acute myeloid leukemia. *Journal of Translational Medicine*.

[32] Yang P., Ni F., Deng R. Q. (2015). miR-362-5p promotes the malignancy of chronic myelocytic leukaemia via down-regulation of GADD45*α*. *Molecular Cancer*.

[33] Ni F., Gui Z., Guo Q. (2016). Downregulation of miR-362-5p inhibits proliferation, migration and invasion of human breast cancer MCF7 cells. *Oncology Letters*.

[34] Mohr A. M., Mott J. L. (2015). Overview of microRNA biology. *Seminars in Liver Disease*.

[35] Pinzón N., Li B., Martinez L. (2017). MicroRNA target prediction programs predict many false positives. *Genome Research*.

[36] Luo D., Zhang Z., Zhang Z. (2018). Aberrant expression of miR-362 promotes lung cancer metastasis through downregulation of Sema3A. *Journal of Immunology Research*.

[37] Neufeld G., Mumblat Y., Smolkin T. (2016). The semaphorins and their receptors as modulators of tumor progression. *Drug Resistance Updates*.

[38] Staton C. A., Shaw L. A., Valluru M. (2011). Expression of class 3 semaphorins and their receptors in human breast neoplasia. *Histopathology*.

[39] Mishra R., Thorat D., Soundararajan G. (2015). Semaphorin 3A upregulates FOXO 3a-dependent MelCAM expression leading to attenuation of breast tumor growth and angiogenesis. *Oncogene*.

