# Supplementary material for: Comparing MicroRNA Profilings of Purified HER-2-Negative and HER-2-Positive Cells Validates miR-362-5p/Sema3A as Characteristic Molecular Change in Triple-Negative Breast Cancers
Source: Dis Markers. 2019 Dec 16;2019:6057280. doi: 10.1155/2019/6057280 (PMC6935799; doi:10.1155/2019/6057280)
Supplement: Supplementary Materials — Table S1: the primers used for real-time PCR. [file 6057280.f1.docx]

Supplemental table1. The primers used for real-time PCR

| Symbols | Forward primer | Reverse primer |
| --- | --- | --- |
| miR-20a-5p | GCGCTAAAGTGCTTATAGTGCA | GTGCAGGGTCCGAGGT |
| miR-221-3p | GGGAAGCTACATTGTCTGC | CAGTGCGTGTCGTGGAGT |
| miR-362-5p | GTCACGAAATCCTTGGAACCTAG | TATGGTTGTTCTCGTCTTCTTCTC |
| miR-502-3p | ACACTCCAGCTGGGAATGCACCTGGGCAAGGA | CTCAACTGGTGTCGTGGA |
| miR-222-3p | GGGGAGCTACATCTGGCT | TGCGTGTCGTGGAGTC |
